# Supplementary material for: Content-rich biological network constructed by mining PubMed abstracts
Source: BMC Bioinformatics. 2004 Oct 8;5:147. doi: 10.1186/1471-2105-5-147 (PMC528731; doi:10.1186/1471-2105-5-147)
Supplement: Additional File 2 — The original results of the above study (non-essential files are deleted to keep the file size under the limit set by BMC bioinformatics). [file 1471-2105-5-147-S2.bz2 › chilibotAdditionalFile2/dip50/397ID8663535E9951/html/EGFR_AREG.html]

 


 **EGFR** and **AREG** 
  
Found 112 abstracts in PubMed, retrieved 50.  
 

 What does Google say? 
 PDF only 
| .edu only 

---

**Interactive relationship** (e.g. stimulation, inhibition, etc)

**Neutral relationship**- We found that amphiregulin  [ **AREG** ]  AR was the most abundant  **EGFR**  ligand expressed in HCA 7 and Caco 2 cells.  Ref: 10362109 Br J Cancer, 1999
- Treatment with either TGFalpha or amphiregulin  [ **AREG** ]  resulted in higher c erbB1  [ **EGFR** ]  activation and c erbB3 levels.  Ref: 12202942 Urol Res, 2002
- These cells express both transforming growth factor alpha and amphiregulin  [ **AREG** ]  and require autocrine signaling through the  **EGFR**  for proliferation and migration.  Ref: 10339571 Proc Natl Acad Sci U S A, 1999
- ... the activation of  **EGFR**  in response to smoke involves cleavage of amphiregulin  [ **AREG** ]  by ADAM 17.  Ref: 12568494 Novartis Found Symp, 2002
- Expression of amphiregulin  [ **AREG** ]  and epidermal growth factor receptor  [ **EGFR** ]  in human breast cancer analysis of autocriny and stromal epithelial interactions.  Ref: 11523048 J Pathol, 2001
- Heparan sulfate proteoglycans and the tetraspanin family of membrane associated proteins appear to act as cofactors in amphiregulin  [ **AREG** ]  driven mitogenesis mediated by the epidermal growth factor receptor  [ **EGFR** ] , but amphiregulin  [ **AREG** ]  s immunolocalization to keratinocyte nuclei and to filopodia may indicate other potentially novel effects.  Ref: 9804327 J Invest Dermatol, 1998
- In support of this, transforming growth factor alpha, EGF and amphiregulin  [ **AREG** ]  mRNAs were detected by RT PCR in the epidermal growth factor receptor  [ **EGFR** ]  expressing cell lines.  Ref: 11875715 Br J Cancer, 2002
- The epidermal growth factor receptor  [ **EGFR** ]   **EGFR**  mediates the actions of a family of bioactive peptides that include epidermal growth factor EGF and amphiregulin  [ **AREG** ]  AR.  Ref: 10085134 J Biol Chem, 1999
- Multiple epidermal growth factor receptor  [ **EGFR** ]  ligands have been identified, including transforming growth factor alpha, heparin binding epidermal growth factor, amphiregulin  [ **AREG** ] , and betacellulin.  Ref: 9655251 Mol Carcinog, 1998
- In comparison, pancreatic carcinoma cell lines commonly demonstrated overexpression of  **EGFR** , erbB2, TGF alpha, Met HGFR, VEGF, and KGF, but they consistently showed marked down regulation of amphiregulin  [ **AREG** ]  mRNA expression.  Ref: 9665487 Am J Pathol, 1998
- The binding of amphiregulin  [ **AREG** ]  and its homolog, heparin binding epidermal growth factor like growth factor, to the receptor is potentially amplified by autoinduction and cross signaling through epidermal growth factor related polypeptides and by transmodulation of other ErbB  [ **EGFR** ]  family receptors HER, 4 in cells expressing these receptors.  Ref: 9804327 J Invest Dermatol, 1998
- AG1478 decreased the expression of genes for amphiregulin  [ **AREG** ] , HB EGF and betacellulin, showing that  **EGFR**  activation induces up regulation of the  **EGFR**  ligands.  Ref: 11133810 Carcinogenesis, 2000
- Amphiregulin  [ **AREG** ]  increased expression of c erbB1  [ **EGFR** ] .  Ref: 12202942 Urol Res, 2002
- RESULTS Oxidative stress and osmotic stress induced tyrosine phosphorylation of  **EGFR**  within 2 minutes, followed by a marked increase in HB EGF and amphiregulin  [ **AREG** ]  transcripts in RGM1 cells.  Ref: 11208719 Gastroenterology, 2001

**Non-interactive relationship** (e.g. studied together, co-existance, homology, etc.)

- Consistent with these profiles, recent gene knockouts established that  **EGFR**  and its ligand, Amphiregulin  [ **AREG** ]  AR, are essential for ductal morphogenesis in the adolescent mouse and likely provide the required epithelial stromal signal.  Ref: 11467454 J Mammary Gland Biol Neoplasia, 2001
- In this study, we compared expression levels of messenger RNAs mRNAs encoding the epidermal growth factor EGF receptor related receptor tyrosine kinases ErbB1  [ **EGFR** ]  through 4, the six EGF receptor ligands, EGF, transforming growth factor TGF alpha, amphiregulin  [ **AREG** ]  ARG, HB EGF, betacellulin, and epiregulin, and the related molecule heregulin alpha, in a series of 10 prostate tissue specimens.  Ref: 10579352 Endocrinology, 1999
- The molecules were localized by double immunofluorescence labeling to detect amphiregulin  [ **AREG** ]  and either the epidermal growth factor receptor  [ **EGFR** ]  or CD44, and the immunostained products were imaged by scanning laser confocal microscopy.  Ref: 9542658 In Vitro Cell Dev Biol Anim, 1998
- There were no significant associations with cytoplasmic or membrane immunoreactivity, but cases showing nuclear expression in >25% of cells were associated with good histological grade, epidermal growth factor receptor  [ **EGFR** ]  expression, c erbB  [ **EGFR** ]  3 positivity, cripto, amphiregulin  [ **AREG** ] , and transforming growth factor alpha overexpression.  Ref: 10749108 Cancer Res, 2000
- The binding of amphiregulin  [ **AREG** ]  to  **EGFR**  then stimulates proliferation of lung epithelial cells.  Ref: 12711607 J Biol Chem< MedlineTA>J Biol Chem, 2000
- The expression of three other ligands for  **EGFR**  epidermal growth factor, cripto, and amphiregulin  [ **AREG** ]  was examined by Northern analysis to determine whether they might also contribute to a potential growth stimulatory loop.  Ref: 9815714 Clin Cancer Res, 1997
- Amphiregulin  [ **AREG** ]  AR and its receptor, epidermal growth factor receptor  [ **EGFR** ]   **EGFR** , were evaluated by dual immunostaining in a series of 84 invasive ductal breast carcinoma specimens, 33 of which were from locally advanced inflammatory T4d cancer.  Ref: 11523048 J Pathol, 2001
- Similarly,  **EGFR**  TK inhibition by ZD1839 reversed the invasive phenotype promoted by the constitutively activated form of the  **EGFR**  EGFRvIII and the  **EGFR**  agonists transforming growth factor alpha TGFalpha, amphiregulin  [ **AREG** ]  and EGF.  Ref: 12881705 Oncogene, 2003
- The presence of epidermal growth factor receptors  **EGFR**  and of its ligands TGFalpha and amphiregulin  [ **AREG** ]  in breast cancer tissues suggests that they play a paracrine autocrine role in tumor growth or progression.  Ref: 9724102 Int J Cancer, 1998
- Topography of amphiregulin  [ **AREG** ]  expression in cultured human keratinocytes colocalization with the epidermal growth factor receptor  [ **EGFR** ]  and CD44.  Ref: 9542658 In Vitro Cell Dev Biol Anim, 1998
- We demonstrate that mRNAs for the 6 major c erbB  [ **EGFR** ]  ligands, namely, epidermal growth factor EGF, transforming growth factor alpha TGF alpha, betacellulin BTC, heparin binding epidermal growth factor like growth factor HB EGF, amphiregulin  [ **AREG** ]  AR and heregulin HRG, are expressed in a large panel of HNSCC cell lines.  Ref: 11072245 Int J Cancer, 2000
- This cell line was shown to have a higher expression of EGF receptors  **EGFR**  and amphiregulin  [ **AREG** ]  AR when compared to their normal counterparts or less aggressive transformed cells.  Ref: 10597254 Oncogene, 1999
- The smoke triggered mechanism includes the generation of oxygen radicals, which in turn stimulate tumor necrosis factor alpha converting enzyme TACE, ADAM 17 metalloproteinase to cleave transmembrane amphiregulin  [ **AREG** ] , a ligand for the epidermal growth factor receptor  [ **EGFR** ]   **EGFR** .  Ref: 12711607 J Biol Chem< MedlineTA>J Biol Chem, 1999
- The intercellular localizations of CD44 with amphiregulin  [ **AREG** ]  and of amphiregulin  [ **AREG** ]  with the epidermal growth factor receptor  [ **EGFR** ]  were strongly concordant.  Ref: 9542658 In Vitro Cell Dev Biol Anim, 1998
- Pervanadate induced, metalloprotease dependent cleavage of the ErbB  [ **EGFR** ]  4 receptor and amphiregulin  [ **AREG** ] .  Ref: 9685416 J Biol Chem, 1998
- Little is known about the regulative role of the four other widely expressed epidermal growth factor receptor  [ **EGFR** ]  ligands, heparin binding EGF, amphiregulin  [ **AREG** ] , betacellulin and cripto in the gastric mucosa.  Ref: 11218236 Scand J Gastroenterol, 2001
- Much of the autonomous growth of cultured keratinocytes is attributable to the signaling of amphiregulin  [ **AREG** ] , a heparin binding autocrine growth factor, through the epidermal growth factor receptor  [ **EGFR** ] .  Ref: 9542658 In Vitro Cell Dev Biol Anim, 1998
- Both amphiregulin  [ **AREG** ]  and the epidermal growth factor receptor  [ **EGFR** ]  segregated to a perinuclear distribution and to intercellular contacts.  Ref: 9542658 In Vitro Cell Dev Biol Anim, 1998
- Our results point to the involvement of several ErbB  [ **EGFR** ]  specific ligands amphiregulin  [ **AREG** ]  and neuregulin 1 and enzymes or adaptor molecules PI3K, Src, Shc and Grb7 in the ErbB  [ **EGFR** ]  pathway dysregulation associated with breast cancer.  Ref: 12866037 Int J Cancer, 2003
- RT PCR transcripts of  **EGFR**  ligands EGF, TGF alpha, HB EGF, and amphiregulin  [ **AREG** ]  were detected in both carcinomas and normal mucosa, indicating that autocrine growth stimulation of colorectal carcinomas is mediated by coexpression of EGF receptor ligands and upregulation of EGF receptors.  Ref: 12790326 Recent Results Cancer Res, 2003
- In this study, we examined the effects of EGF, TGFalpha, amphiregulin  [ **AREG** ]  and heregulin alpha HRGalpha on proliferation, wound closure, and the expression of their receptors c erbB1  [ **EGFR** ]  c erbB4 in primary cultures of human urothelial cells in vitro.  Ref: 12202942 Urol Res, 2002
- 5 OH 2D3 did not affect the levels of the proteoglycan dependent  **EGFR**  ligands amphiregulin  [ **AREG** ]  and heparin binding EGF nor the synthesis of proteoglycans, as assessed by 35S labeling and ion exchange chromatography.  Ref: 9927298 Endocrinology, 1999
- These epithelial cells are derived from a normal human breast epithelial cell culture transformed by SV40 T Ag, are of the same clonal origin, have respectively increasing levels of  **EGFR** , TGFalpha, amphiregulin  [ **AREG** ]  and of thymidine kinase activity associated with increasing tumorigenic potential in nude mice tumor intake and tumor volume.  Ref: 9724102 Int J Cancer, 1998
- We conclude that amphiregulin  [ **AREG** ]  acts as an autocrine growth factor in HCA 7 and Caco 2 cells, and  **EGFR**  ligand induced proliferation is influenced by cellular polarity.  Ref: 10362109 Br J Cancer, 1999
- Reverse transcription polymerase chain reaction was performed on each sample with primers for rat epidermal growth factor, epidermal growth factor receptor  [ **EGFR** ]  ErbB  [ **EGFR** ] , heparin binding epidermal like growth factor, hepatocyte growth factor, hepatocyte growth factor receptor, keratinocyte growth factor, betacellulin, amphiregulin  [ **AREG** ] , and neuregulin alpha.  Ref: 12148846 Laryngoscope, 2002
- Up regulation of transmembrane tyrosine kinase receptors c met, IGFR1, IGFR2 and  **EGFR**  family related ligands such as HB EGF, TGFa, amphiregulin  [ **AREG** ]  and neuregulin as well as c met ligand HGF point to a possible role of this system in metastasis.  Ref: 12174879 Anticancer Res< MedlineTA>Anticancer Res, 2002
- **EGFR**  mRNA was detected in all tumours, transforming growth factor alpha TGFalpha mRNA levels were similar to those in normal bladder urothelia or were decreased and mRNA levels for amphiregulin  [ **AREG** ] , heparin binding epidermal growth factor like factor HB EGF and betacellulin were significantly higher than those in normal urothelia.  Ref: 11133810 Carcinogenesis, 2000
